# Capacitive Control of Spontaneously Induced Electrical Charge of Droplet by Electric Field-Assisted Pipetting

**DOI:** 10.1007/s40820-015-0048-2

**Published:** 2015-06-20

**Authors:** Horim Lee, Dongwhi Choi, Dong Sung Kim, Geunbae Lim

**Affiliations:** 1 Department of Mechanical Engineering, Pohang University of Science and Technology (POSTECH), 77 Cheongam-ro, Pohang, Gyeongbuk 790-784 South Korea; 2 Analysis Science & Engineering Team, Semiconductor R&D Center, Samsung Electronics Co., Ltd., Samsungjeonja-ro, Hwaseong, Gyeonggi 445-701 South Korea

**Keywords:** Pipet tip, Capacitive control, Electrical charge, Self-assembly

## Abstract

The spontaneously generated electrical charge of a droplet dispensed from conventional pipetting is undesirable and unpredictable for most experiments that use pipetting. Hence, a method for controlling and removing the electrical charge needs to be developed. In this study, by using the electrode-deposited pipet tip (E-pipet tip), the charge-controlling system is newly developed and the electrical charge of a droplet is precisely controlled. The effect of electrolyte concentration and volume of the transferred solution to the electrical charge of a dispensed droplet is theoretically and experimentally investigated by using the equivalent capacitor model. Furthermore, a proof-of-concept example of the self-alignment and self-assembly of sequentially dispensed multiple droplets is demonstrated as one of the potential applications. Given that the electrical charge of the various aqueous droplets can be precisely and simply controlled, the fabricated E-pipet tip can be broadly utilized not only as a general charge-controlling platform of aqueous droplets but also as a powerful tool to explore fundamental scientific research regarding electrical charge of a droplet, such as the surface oscillation and evaporation of charged droplets.

**Electronic supplementary material:**

The online version of this article (doi:10.1007/s40820-015-0048-2) contains supplementary material, which is available to authorized users.

## Introduction

A pipet tip is a common laboratory tool for transporting a desired volume of liquid in many scientific and engineering fields, such as chemistry, biology, and medicine. We have recently reported that the conventional pipetting spontaneously induces a considerable amount of electrical charge (order of 0.1 nC) depending on the constituents of the dispensed droplet and the coating material of the pipet tips [1]. This spontaneous droplet electrification is due to the contact between the aqueous liquid and the inner surface of the polymeric pipet tip during the pipetting process called solid–liquid contact electrification. Nowadays, with its own power generation nature of the contact electrification, the various applications of the solid–liquid contact electrification have been reported such as energy harvesters from the natural water motion and sustainable self-powered sensors [2–4]. However, solid–liquid contact electrification causes negative effects during the pipetting procedure, such as a difficulty in detachment of tiny droplet from pipet tips, increase of surface tension of aqueous droplet, and modification of chemical characteristics of solutions owing to its considerable amount of electrical charge [5–7]. These effects are mostly undesirable and unpredictable for most experiments by using pipetting and can further cause unexpected problems in specific cases, such as the coalescence, evaporation, and surface oscillation of droplets [1, 7–11]. Thus, a method for controlling and removing the electrical charge of the droplet dispensed by conventional pipetting needs to be developed.

The controlling method of the electrical charge of the droplet to be developed should meet the following requirements: (i) the method must not hinder a pipetting procedure to transfer an accurate volume of liquid; (ii) the method must not cause the change of the constituents of the liquid to be transferred. The methods that meet these requirements can be divided into two: the passive and active methods. The passive method uses no external energy but controls the solid–liquid contact electrification phenomenon itself. The use of less ionizable material for coating the inner surface of the pipet tips reduces the electrical charge of the droplet because the origin of the electrical charge is the ionization of the inner surface of the pipet tip. In this point of view, the graphene-nanocomposite-coated pipet tip called the zeta (ζ)-pipet tip is fabricated and reported to reduce the spontaneously induced electrical charge of the dispensed aqueous droplet from a pipet tip [12]. In the report, the graphene nanocomposite that has lower zeta potential than the inner surface material of a conventional pipet tip was used as the proper coating material. To control the electrical charge of the droplet, along with the use of less ionizable material, the contact area between the aqueous solution and pipet tip inner surface can also be controlled by changing the geometric design of the pipet tips because the contact area is one of the key parameters on the contact electrification phenomenon. The passive method has several advantages: it does not need external energy and has a simple fabrication process. However, the selection of the proper coating material or the modification of the geometry design of the pipet tip inner surface will be limited for accurate transferring and easy detachment without leaving a residual droplet in the pipet tips. Particularly, the complete removal of the electrical charge cannot be achieved by using the passive method. By contrast, the active method uses external electricity to remove or control the electrical charge of the droplet; this method is also called the capacitive charging method. Applying an external electric field to the aqueous solution inside the pipet tip induces an additional electrical charge with the same mechanism of the capacitor. If the desired amount of the additional charges to the aqueous solution inside the pipet tip can be precisely induced, the resultant total amount of the charge of the dispensed droplet can be easily controlled. For example, by inducing the same magnitude with the opposite sign of the electrical charge to the solution, the droplet to be dispensed will be neutralized and enable the complete removal of the electrical charge from various liquids.

In this work, the capacitive charging method is applied to control and remove the electrical charge during the pipetting procedure. The electrode-deposited pipet tip (E-pipet tip) is fabricated to apply the external electric field to the solution inside the pipet tip. By applying the proper voltage, the electrical charge of the dispensed droplet can be easily controlled. The effect of the applied voltage to the resultant electrical charge of the droplet is investigated to characterize its performance. Furthermore, the zero charge voltage (ZCV), which is the proper applied voltage for the complete removal of the electrical charge of the droplet, is explored by varying the electrolyte concentration and volume of the transferred solution. As a proof-of-concept application, the self-alignment and self-assembly of sequentially dispensed multiple droplets with intentionally controlled electrical charges are demonstrated by using the fabricated E-pipet tip. Given that the electrical charge of the various aqueous droplets can be precisely and simply controlled, the fabricated E-pipet tip can be broadly utilized as a general charge-controlling platform of aqueous droplets.

## Materials and Methods

### Materials

All KCl solutions were prepared by using deionized water (SAMCHUN Chemical, Korea). In self-alignment and self-assembly of sequentially dispensed multiple droplet experiments, highly viscous silicone oil (Shin-Etsu, KF96 50 cSt) was used as the surrounding medium to make aqueous droplets moving in the low Reynolds number regime.

### Electrical Charge Measurement

The Faraday cup method was used to measure the amount of the electrical charge of a droplet. The charge was directly measured by using a laboratory-made Faraday cup connected to an electrometer (Keithley Model 6517A). The Faraday cup is composed of two cylindrical electrodes separated by an insulator. When the charged droplet was dispensed into the inner electrode, the counter charge was transferred to the inner electrode from the electrometer to satisfy electrical neutrality. The total charge was measured by integrating the current from the electrometer.

### Fabrication of E-pipet Tip

As an active electrode, conductive epoxy (silver paste, Circuitworks™) was coated on a conventional disposable pipet tip. To avoid direct contact with the liquid, the edge of the tip was deliberately opened by using a 4-mm masking tape. An electric wire was then coiled around the outside of the tip. The tip was turned upside down and was covered with the conductive epoxy. The conductive epoxy was diluted with isopropyl alcohol to uniformly and completely cover the outer wall of the pipet tips by lowering its viscosity. Thereafter, the tips were placed in an oven at 65 °C for 30 min to cure the epoxy. After curing, the masking tape was removed from the tips. The exposed conductive epoxy was also covered with dielectric epoxy (PERMA™ Epoxy) for insulation. To prevent the epoxy from flowing down, it was left in the air for 5 min before covering.

### Electrical Charge Control Experiment

Two electrodes, platinum wire and active electrode, were used to create a capacitive charge in liquid to be transferred. A platinum wire was placed in a reservoir of solution. An electric voltage was applied to the active electrode which was deposited on the outer surface of conventional pipet tips during aspiration of liquid. After aspirating the solution, the pipet tip is isolated from the reservoir. Then, the applied voltage turned off and the solution is dispensed from the pipet tip.

## Results and Discussion

**Fig. 1:**
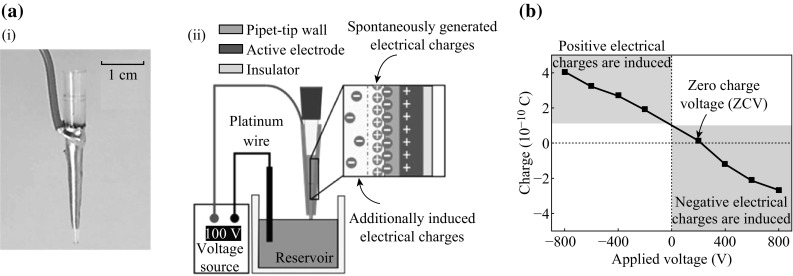
**a** Schematic of the fabricated E-pipet tip and charge-controlling systems. **b** The result of the electrical charge measurement experiments with varying the applied voltage on the active electrode of the E-pipet tip

As shown in Fig. 1a, an E-pipet tip is fabricated to control the electrical charge of the dispensed droplet by applying the external electric voltage to the pipet tip outer surface with a generated electric field on the solution inside the pipet tip. By using the E-pipet tip, the charge-controlling system is developed and the operating procedure is as follows. First, the E-pipet tip, reservoir, and voltage source are all electrically connected with the electric wire. After equipping the E-pipet tip with a conventional pipet, it is immersed into the reservoir to aspirate the solution. After the solution is aspirated, the solid–liquid contact electrification occurs at the interface between the pipet tip inner surface and the solution, thus resulting in the generation of electrical charge pairs into an electric double layer (EDL). The amount and sign of the electrical charges are strongly related with various properties of the solution and pipet tip inner surface material, such as electrolyte concentration, pH of the solution, and zeta potential of the pipet tip inner surface material [1, 12–14]. If negative (positive) electrical charges are generated on the pipet tip inner surface, then positive (negative) electrical charges will be generated on the solution nearby the interface. To induce additional electrical charges to the solution inside the pipet tip, the electric voltage is applied to the active electrode deposited on the pipet tip outer surface through the voltage source. When the positive voltage is applied on the electrode as shown in Fig. 1a, the positive charges will be on the electrode, thus resulting in generation of spreading electric fields from the electrode to the solution through the pipet tip. The electric fields attract the negative charges from the reservoir to the solution nearby the interface; this phenomenon causes electrical neutrality. By maintaining applied electric voltage, the E-pipet tip is removed from the reservoir and the solution inside the pipet tip is physically and electrically isolated from the reservoir. Thereafter, the applied voltage is removed by turning off the voltage source. The induced additional electrical charges play an important role in controlling the total amount of electrical charge of the dispensed droplet because the electrical charges inside the isolated solution are swept with the solution during dispensing process. By varying the applied voltage from −800 to 800 V, the electrical charge measurement experiments with 7 µL deionized (DI) water droplets are performed. Its result is plotted in Fig. 1b. The electrical charge amount with no applied voltage is about 1 × 10^−10^ C, which is similar with the electrical charge of water droplet as previously reported [1, 12]. Applying negative (positive) voltage to the electrode, the additional positive (negative) electrical charges are induced on the solution inside the pipet tip, as previously explained. Notably, a ZCV appears, at which the electrical charge of a dispensed droplet becomes zero representing the complete removal of electrical charge of the droplet, which could be obtained from the points of intersection of the charge-applied voltage curves with the x-axis line.

**Fig. 2:**
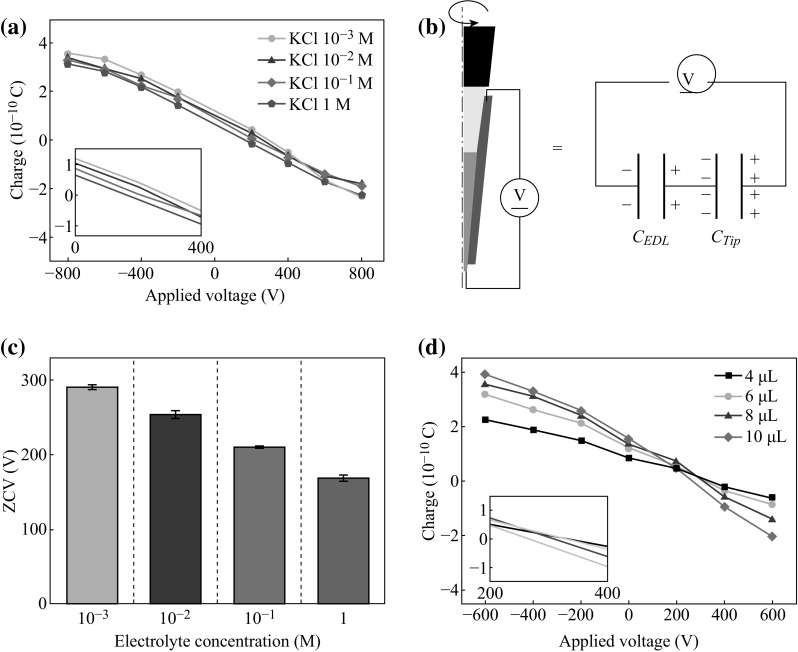
**a** Voltage dependence of the electrical charge of a dispensed droplet for various electrolyte concentrations. **b** Equivalent circuit of E-pipet tip system with applied voltage. **c** The ZCVs for various electrolyte concentrations. **d** The voltage dependence of the electrical charge of a dispensed droplet with various volumes of the transferred solution. The *inset* shows the magnified curves near ZCV

The amount of the electrical charge on the droplets dispensed from a pipet tip is strongly dependent on the electrolyte concentration [1, 12]. To investigate the effects of electrolyte concentration and applied voltage on the electrical charge of dispensed droplet, the electrical charge measurement experiment is performed by changing the electrolyte concentration from 1 mM to 1 M as shown in Fig. 2a. The amount of the electrical charge is almost negatively proportional to the applied voltage and the curves are parallel to each other regardless of the electrolyte concentration (Fig. 2a, inset). This fact can be explained by using equivalent capacitor model. Electrolyte solution can be considered as a perfect conductor because a constant voltage is applied in the aspiration and electrical relaxation time of the system is assumed to be very short [15]. Thus, this system can be simply represented by a series of two capacitors, namely, EDL and pipet tip wall capacitors as shown in Fig. 2b [16–18]:

1 $$C_{\text {Total}} = \frac{{C_{\text {Tip}} \times C_{\text {EDL}} }}{{C_{\text {Tip}} + C_{\text {EDL}} }},$$

where *C*_Total_, *C*_Tip_, and *C*_EDL_ are total capacitance of the E-pipet tip system, capacitance of the pipet tip wall, and capacitance of the EDL, respectively. Furthermore, *C*_Tip_ and *C*_EDL_ could be described as follows:

2 $$C_{\text {tip}} = \varepsilon_{\text {tip}} \frac{A}{t}\,\,{\text{and}}\,C_{\text {EDL}} = \varepsilon_{\text {water}} \frac{A}{{\lambda_{D} }},$$

where *ε*_tip_, *A*, *t*, *ε*_water_, and *λ*_D_ are the permittivity of the pipet tip wall (order of 1), contact area between the solution and pipet tip inner surface, thickness of the pipet tip wall (order of 10^−3^ m), permittivity of the water (order of 10), and debye length (order of 10^−9^ m), respectively. In Eq. 2, *C*_EDL_ (order of 10^10^ × *A*) is larger than *C*_Tip_ (order of 10^3^ × *A*). Thereby, *C*_Total_ can be simplified to *C*_Tip_ according to Eq. (1). Consequently, Eq. (1) represents that the concentration of the liquid, wherein *C*_EDL_ is dependent, does not affect the total capacitance and the amount of the capacitive charge. The electrical charge of the droplet with lower concentration stays above that of the droplet with the higher concentration under almost all conditions (Fig. 2a). This result is due to the difference of the surface ionization corresponding to the concentration [1, 14]. Higher ZCV is needed to completely remove the electrical charge of the dispensed droplet for lower concentration solution (inset of Fig. 2a, c) because the spontaneously generated electrical charge owing to the surface ionization becomes more positive with decreasing concentration [1].

The voltage dependence of the electrical charge is also influenced by the volume of the transferred solution. As shown in Fig. 2d, when the volume of the transferred solution is larger, the inclined angle of the curve becomes more negative; i.e., the controlling performance of the capacitive charging method becomes higher. Voltage dependence can be simply explained with the ratio of the area where the capacitive charging occurs (*A*_*a*_) to the total contact area (*A*_*t*_) of the solution with the inner surface of the pipet tip (*R* = *A*_*a*_/*A*_*t*_). *R* indirectly represents the controlling performance of the capacitive charging method to the total E-pipet tip system. Given that the edge of the pipet tip is not treated by the deposition of active electrode to not hinder the detachment of a droplet from the tip (Fig. 1a), *R* becomes larger with the increasing volume of the transferred solution, thus implying a higher effect than in the case of the smaller volume of the transferred solution. As a result, when the transferred volume of solution becomes smaller, a higher ZCV is required with decreasing *R* to compensate for the effect of opened surface. If the active electrode is deposited on the whole surface of the pipet tip and the surface charge density owing to the solid-water contact electrification is ideally uniform, the ZCV will be constant regardless of the transferred volume of the solution.

**Fig. 3:**
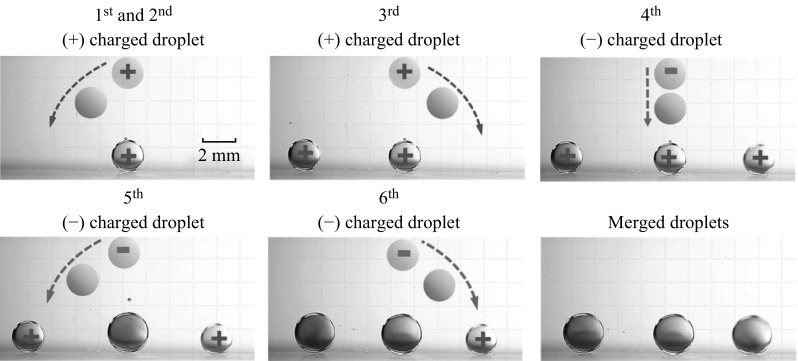
Proof-of-concept demonstration of self-alignment and self-assembly of sequentially dispensed droplets. To visualize the sign of the droplet charge, *red* and *green* dyes are used to represent the *positive* and *negative* signs, respectively. (Supporting video V1). (Color figure online)

By using the developed charge-controlling system, the electrical charge of the dispensed droplet can be easily and precisely controlled by adjusting the applied voltage (Fig. 2). In this work, simple self-alignment and self-assembly of sequentially dispensed multiple droplets are demonstrated by using the developed charge-controlling system as a proof-of-concept application. The first two pictures of Fig. 3 show the behavior of three positively charged DI water droplets with negative applied voltages of −300 V consecutively dispensed at the middle position. The amount of the electrical charge of the droplet is about 0.2 nC. The droplets repel each other owing to the electrostatic repulsion; hence, they move away from each other although they are dispensed at the same position. This result represents the self-alignment of droplets. After the self-alignment of droplets, three negatively charged droplets with positive applied voltage of 600 V are consecutively dispensed (Fig. 3). The amount of the electrical charge of the droplet is about −0.2 nC, which is same magnitude but opposite sign with the positively charged droplet above. The first negatively charged droplet is attracted and merged with the positively charged droplet in the middle position because the attractive forces toward the other droplets are balanced and the droplet experiences stronger attractive force toward the positively charged droplet at the middle position. After two oppositely charged droplets are merged, the merged droplet becomes neutralized. The second negatively charged droplet is merged with the left-side positively charged droplet because this droplet is dispensed at a position slightly closer to the left side. Thereafter, the third negatively charged droplet only interacts with the right-side positively charged droplet because the other droplets are already neutralized (Supporting video V1), that is, the systematic self-assembly of droplets is achieved by the electrical interaction among them. Consequently, the self-alignment and self-assembly of the droplets are easily performed by only altering the sign of electrical charge of the droplet without any additional operations, which could be exploited in the droplet-based experiments particularly for sequential multi-step droplet merging.

## Conclusion

In summary, E-pipet tip is fabricated to precisely control the electrical charge of a dispensed droplet by the active controlling method. The charge-controlling system is developed and the electrical charge of the dispensed droplet is controlled by using the E-pipet tip. The controlling behavior and the ZCV of the system are systematically studied by using an equivalent capacitor model. Furthermore, the charge-controlling system is used to demonstrate the self-alignment and self-assembly of sequentially dispensed multiple droplets as one of the applications. The developed system enables easy control of electrical charge of a droplet and would be used to explore fundamental scientific research, such as the surface oscillation and evaporation of charged droplets.

## Electronic supplementary material

Below is the link to the electronic supplementary material.

Supplementary material 1 (AVI 3114 kb)
